# Cost-utility of stereotactic radiation therapy *versus* proton beam therapy for inoperable advanced hepatocellular carcinoma

**DOI:** 10.18632/oncotarget.17369

**Published:** 2017-04-21

**Authors:** Henry W.C. Leung, Agnes L.F. Chan

**Affiliations:** ^1^ Department of Radiation Oncology, An Nan Hospital, China Medical University, Tainan, Taiwan; ^2^ Department of Nursing, Min-Hwei College of Health Care Management, Tainan, Taiwan; ^3^ Department of Pharmacy, An Nan Hospital, China Medical University, Tainan, Taiwan

**Keywords:** proton beam, SBRT, ICER, NMB, WTP

## Abstract

The cost-utility of proton beam therapy was compared to stereotactic body radiation therapy for inoperable advanced hepatocellular carcinoma. A Markov decision-analytic model was performed following time to progression and survival using phase II trial data. Patients transitioned between three health states. Clinical outcomes were estimated for quality of life using utility estimates in the published literature and measured as incremental cost-effectiveness ratios (ICERs) and net monetary benefits (NMBs). Real direct medical costs were extracted from the Bureau of National Health Insurance database. One-way and probabilistic sensitivity analyses assessed the impact of specific variables on the model. In the base-case scenario, the modeled median survival was 16 months for proton beam therapy and 10 months for SBRT. Proton beam therapy resulted in an additional 2.61 quality-adjusted life years (QALYs) at an incremental cost of NT$ 557,907 compared to SBRT. The ICER was NT$ 213,354 per QALY gained. The probabilistic sensitivity analysis predicted a 97 % chance of proton beam therapy being cost-effective at the willingness to pay NT$2,157,024 per QALY gained. Thus, proton beam therapy is a cost-effective therapy for inoperable advanced hepatocellular carcinoma at the willingness-to-pay threshold of Taiwan.

## INTRODUCTION

Approximately 80% of all liver cancers are hepatocellular carcinoma (HCC), a primary malignant neoplasm derived from hepatocytes. HCC was the leading cause of cancer death worldwide in 2012, and the second leading cause of cancer death in Taiwan in 2014 [1, 2]. The HCC incidence rate is higher in men than in women and nearly 50% of all cases and deaths are reported in China [3]. The 5-year survival rate for patients diagnosed with HCC is exceedingly poor at 3-5% [4]. For inoperable advanced HCC (mHCC), the 1-year median survival rate is 20-30 % [5-6].

For patients with small inoperable HCC (≤5 cm diameter), local ablative treatments, such as transcatheter arterial chemoembolization (TACE) and radiofrequency ablation, achieve excellent local control [7]. However, treatment of large HCC (≥7 cm diameter) is still challenging, as no standard treatment strategy is available. Modern radiation therapy includes stereotactic body radiotherapy (SBRT) which has been used for large mHCC [8-12]. A prolonged median overall survival of 17 months and 31 months was reported by two phase II clinical trials [8, 9]. However, the use of these modern radiation therapies has high treatment costs, though no study has assessed whether the clinical benefits may offset the increased cost. The objective of the present study was to compare the cost-utility of PBT and SBRT for patients with mHCC from the perspective of a single payer healthcare system.

## MATERIALS AND METHODS

### Clinical data sources

We performed a systematic literature search of the PubMed database to identify all randomized controlled trials (RCTs) of PBT or SBRT for inoperable advanced HCC performed from January 1, 1999, to September 31, 2016. The search strategy was based on combinations of (“unresectable” or “inoperable” or “advanced” or “metastatic” hepatocellular carcinoma” [Mesh]) and (SBRT or proton [Mesh] ) (“randomized controlled trials” or” clinical trials” [Mesh]). We also searched cost-effectiveness studies using the medical subject headings or key words: quality-adjusted, QALY, life-year gained, and cost-effectiveness. Two reviewers (AC and HL) were responsible for independently evaluating the appropriate full text with reference to the same inclusion and exclusion criteria. RCTs or clinical studies published in English regarding the treatment of mHCC by PBT or SBRT were included. Letters to the editor, case reports, non-randomized trials, animal studies, editorials, and posters were excluded. Any discrepancies between reviewers were resolved by consensus. We finally selected one phase I/II RCT of SBRT and one phase II study of PBT for mHCC as the clinical data source for the model.

### Markov model

**Figure 1:**
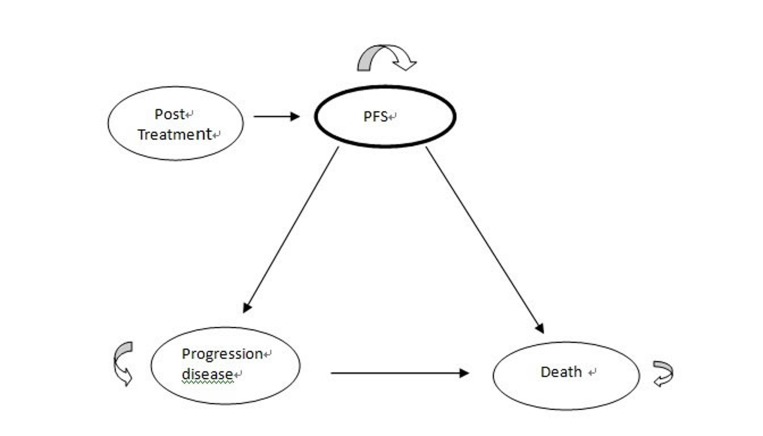
Markov model schema in advanced HCC Ovals presented the differing health states. Arrows indicated pathways that can occur. Arrow returned back to the same health state and remained in that health state.

A Markov model was constructed using TreeAge Pro2014 Suite (R1.0 Released; TreeAge Inc., Williamstown, MA) to evaluate the costs, health outcomes, and cost-effectiveness of PBT versus SBRT in the treatment of mHCC. In the base case analyses, the model simulated a hypothetical cohort of 10,000 patients and repeated 1,000 times for each of the two treatment regimens. The time horizon of the model was 5 years (60 months). We hypothesized three health states: stable disease, disease progression and death in the model according to the phase I/II clinical trials, the phase II trial and the expert opinions [8, 9] (Figure 1). A patient in the model was considered to be in one of the three health states at any time. All patients began in the stable stage and transitioned from one state to another on the basis of the transition probabilities; they received either SBRT or PBT. In the model, we did not include deaths from natural causes that occurred in any health state. Death from cancer was assumed to occur after disease progression. The model perspective was based on the Bureau of National Health Insurance (BNHI) in Taiwan, with a 1-month cycle length adjusted to half-cycle in each health state process. The willingness-to-pay (WTP) threshold was defined by the World Health Organization (WHO) as 3-times the per capita gross domestic product (GDP) [13, 14]. The Taiwan per capita GDP in 2016 was NT$719,008 (US$22,469) [15]; therefore, the WTP threshold was considered to be NT$2,157,024/QALY. A panel of local experts (blood oncologist, radiation oncologist, and one expert in pharmacoeconomic analysis) was consulted to ensure that the assumptions in the model reflected routine clinical practice.

### Treatment regimen

Our treatment schema and outcomes were modeled from the phase I/II SBRT study and phase II PBT study [8, 9]. According to expert opinion, the total radiation dose, patterns of treatment failure, and patient survival were assumed to be the same as in the studies. According to the phase I/II trial, the SBRT treatment regimen doses were 30 to 54 Gy (24 to 54 Gy in Trial 1) in six fractions every other day over 2 weeks, delivered to the planning target volume (PTV). The dose to tumor vascular thrombosis plus PTV margin could be limited to 30 Gy. The PBT regimen had a total dose ranging from 50 Gy in 10 fractions to 87.5 Gy in 30 fractions (median, 72 Gy in16 fractions) and was administered without serious acute and late adverse events. All patients received PBT to a total dose of 76 Gy for 5 weeks in once daily 3.8-Gy fractions four days a week using a 150 to 190 MV proton beam. Detailed treatment was mentioned in two clinical trials. The severe toxicities (≥grade 3) necessitating treatment were considered in our model.

### Probabilities and utilities

The transition probabilities of the health states were estimated using the equation published previously: P(1 month) = 1- (0.5) ^(1/median time to event)^ [16-18]. Health state utilities were reduced according to the incidence rate of the severe adverse event (≥grade 3) reported in the two clinical trials.

### Direct medical costs

The direct health care medical costs were extracted from the BNHI database in 2016, including drug costs, laboratory test, physician visits, pharmacy dispensing fees, and treatment costs for grade 3/4 adverse events. Based on a policy issued by the BNHI, the reimbursement cost for SBRT is approximately NT$213,660 as a treatment package. We also assumed that the PBT was reimbursed at NT$300,000 as a package, which is now paid by the patients for the treatment of mHCC. All costs have been discounted at a real annual rate of 3% to adjust for the relative value of the Taiwan dollar.

### Cost-utility analysis

Cost-utility was evaluated using the incremental cost-effectiveness ratio (ICER) and net monetary benefit (NMB) method. ICERs were calculated as the ratio of the difference in total direct medical costs to the difference in QALYs [19]. The NMB approach was changed in various WTP thresholds. NMB is defined as ΔEλ-ΔC, where λ is the WTP per QALY threshold, ΔE is the effectiveness, and ΔC is the incremental cost of two treatments. NMB regression analyses were modeled to compare SBRT with PBT based on varying the threshold value from NT$0.00 to NT$2,157,024 [20]. When the NMB value of the regimen was positive at a specific WTP value, we hypothesized that the regimen was cost-effective at the WTP value.

### Sensitivity analysis

A tornado diagram was conducted to determine the major parameters impacting the model. The probabilistic sensitivity analysis was performed using a Monte Carlo simulation based on 10,000 samples by varying all parameters over a range of ±30 % in relation to the base-case data in the model simultaneously (Table 2) . The distributions for each parameter in the probabilistic sensitivity analysis were modeled. Log-normal distributions were adopted for all costs, and beta distributions were adopted for probabilities, utilities, and toxicity.

**Table 1:** Estimated cost inputs used in the model

Cost input	Value	
**Costs** (NT$)		
	Proton	SBRT
Treatment cost	300,000	213,660
Costs of laboratory test, CT	0	12982
**Sub-total of PFS stage**	**300,000**	**226,642**
Costs of laboratory test, CT	94000	82,801
Treatment cost for toxicity	6493	63,054
**Sub-total of PD stage per visit**	**100493**	**146,305**
**Total**	**400493**	**372947**

Abbreviation: PFS, progression free survival; PD, progression disease, CT, computerized tomography;

SBRT, stereotactic body radiotherapy

**Table 2:** Parameters value in base-case and ranges in sensitivity analyses (± 30%)

Parameters	Base estimate (3% discount)	Lower- Upper Limit Limit	Assumed Distribution
**Transition Probability**			
ProgToMeta for proton	0.1295	0.09-0.1685	Beta
ProgToDead for proton	0.06697	0.047-0.08697	Beta
PD To death for proton	0.0219	0.015-0.029	Beta
ProgToMeta1 for SBRT	0.109	0.076-0.142	Beta
ProgToDead1 for SBRT	0.08299	0.0581-0.1079	Beta
PD To death1for SBRT	0.0399	0.0279-0.0519	Beta
**Utility**			
PFS for proton	0.399	0.279-0.519	Beta
PD for proton	0.28	0.196-0.476	Beta
PFS1 for SBRT	0.375	0.263-0.488	Beta
PD1 for SBRT	0.263	0.184-0.342	Beta
**Direct Medical Costs (US$=32 NT)**			
cPFS for proton	291000	203700-378300	Constant
cPFS1 for SBRT	219843	153890-285796	Constant
cPD for proton	97478	68235-126721	Constant
cPD1 for SBRT	141916	99341-184491	Constant

Abbreviations: c, cost; SBRT, sterotactic body radiation therapy; PFS, progression free survival; PD, progression disease

All costs presented were discounted at 3% from the original price to adjust for the relative value of the Taiwan dollar.

## RESULTS

### Base-case analysis

**Figure 2:**
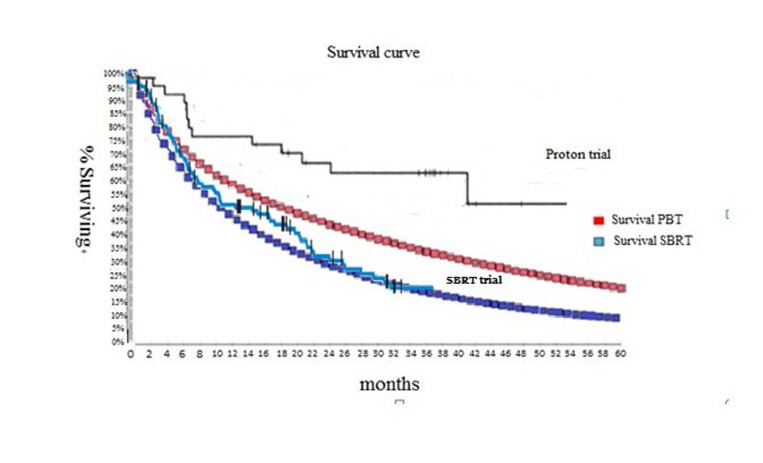
Modeled Kaplan- Meier Analysis of overall survival Trials data were referred to published literature.[8, 9].

Modeled outcomes in terms of median overall survival were consistent with the study target data. The median overall survival at 1 year reported in PBT trial and SBRT trial were 77% and 63% , 2 year were and 66% and 51%, respectively (Figure 2). The patient characteristics retrieved from two trials were similar, and no significant differences were found in sex, age, ECOG performance status, plasma levels of α-fetoprotein (AFP), Barcelona Clinic Liver Cancer stages B and C, percentage of Child-Pugh class A liver function, or tumor size. Patients with underlying hepatitis C and Child-Pugh class A were significantly different between the two trials (Table 3). The incidence of grade 3/4 toxicity was 30% and 34.3 % for PBT and SBRT, respectively.

**Table 3:** Baseline characteristics of the patients in the Phase II and Phase I/II trials

**Characteristics**	**Phase II proton trial**	**Phase I/II trial**	***P* value**
**Age,years**	70	69.4	0.22
**Male no (%)**	20 (67%)	80 (78.4%)	0.287
**Underlying liver disease**			
Hepatitis B	3 ( 10 %)	39 (38.2%)	0.06
Hepatitis C	26 (87%)	39 ( 38.2%)	< 0.0001
**ECOG performance status *n* (%)**			
0	29 ( 97%)	85 ( 83.3)	0.05
1			
2	1 (3 %)	11 ( 10.8%)	0.81
**Child-Pugh class, no (%)**			
A	20 (67 %)	102 ( 100%)	< 0.0001
B	10 (33%)	0 (0%)	
C	67 ( 65.7%)	0 (0%)	
**Tumor Size, median**	45 mm	72 mm	
**Biochemical analysis**			
Albumin (g/dl)	0	4.0	
Total bilirubin ( mg/dl)	0	1.3	
Alpha-fetoprotein	<300 ng/ml	163 nmol/L	
**Previous therapy**			
Local ablation /TACE	11 (37 %)	22 ( 21.6%)	0.368
**Macrovascular invasion ( no,%)**	12 (40 %)	20 (49%)	0.63

Abbreviations: ECOG, Eastern Cooperative Oncology Group; BCLC, Barcelona Clinic Liver Cancer staging system; TACE, transarterial chemoembolization; RFA, radiofrequency ablation; PEI, percutaneous ethanol injection.

The direct health care medical costs per month by disease stage and treatment group are shown in Table 1. PBT had the highest cost for each patient in the stable stage (NT$300,000), whereas the SBRT group experienced the highest cost in the progressive stage (NT$146,305) because of high treatment costs for grade 3-4 toxicity. PBT resulted in an additional 2.61 QALY gains at an incremental cost of NT$557,907. The ICER for PBT versus SBRT was NT$ 213,354 per QALY gained.

### Sensitivity analyses

A tornado diagram illustrated the results of one-way sensitivity analysis that the highly sensitive 6 parameters were the utility of patients treated with PBT or SBRT in stable and progressive states as well as the direct medical costs in both states. The high value for utility of patients treated with PBT in stable and progressive states results in proton being the preferred strategy (Table 4).

**Table 4:** Tornado sensitivity analysis-ICER report

VARIABLE NAME	VARIALE RANGE	LOW VALUE	HIGH VALUE	SPREAD	SPREAD SQR	RISK PCT	CUMUL PCT
uPD	0.196 to 0.476	-3834190.96694	634945.18245	4469136.14939	19973177921776.06	0.19003	0.33323
uPFS	0.279 to 0.519	-1780405.66472	1448835.01484	3229240.67956	10427995366529.27	0.09921	0.14257
uPD1	0.184 to 0.342	-1324462.00957	1684826.81114	3009288.82071	9055819206466.062	0.08616	0.47922
cPFS	203700.0 to 378300.0	-422990.16247	1452808.99214	1875799.15461	3518622468452.824	0.03348	0.04333
uPFS1	0.263 to 0.488	297633.7112	1954325.66909	1656691.95789	2744628243332.1616	0.02611	0.39306
cPD1	99341.0 to184491.0	-233531.45512	1263350.28479	1496881.7399	2240654943250.1396	0.02132	0.36158

Remarks: uPD, uPFS : utility of patients treated with proton in stable states and progressive state; uPD1and uPFS1, utility of patients treated with SBRT in stable states and progressive state; cPFS, direct medical cost for proton in progression state; cPD1, direct medical cost for SBRT in stable state.

**Figure 3:**
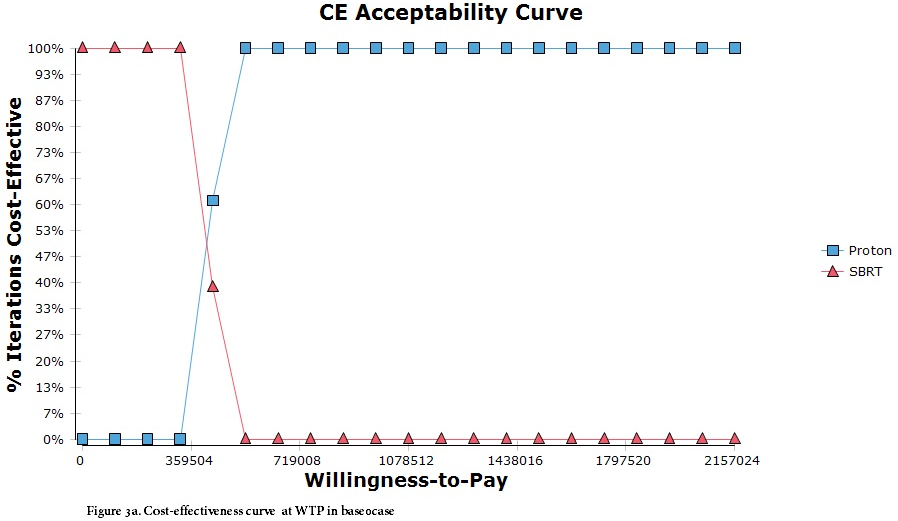

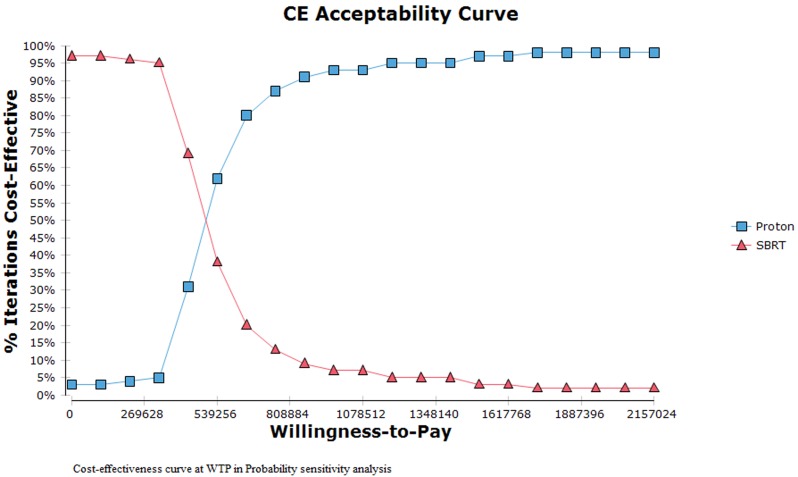
(**Upper**).Cost-effectiveness curve at WTP in base case. (**Lower**). CEA at WTP in PSA.

**Figure 4:**
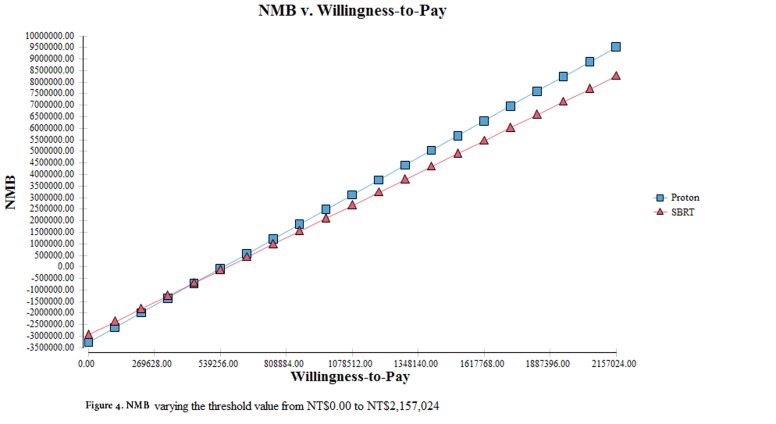
NMB varying the threshold value from NT$ 0.00 to NT$2,157,024

The Monte Carlo simulation demonstrated that the probability of PBT and SBRT being cost-effective was 97% and 4%, respectively, at the WTP threshold of NT$2,157,024 (Figure 3). The more positive NMB values for PBT compared to SBRT by varying the WTP threshold indicated that PBT was likely to be cost-effective at the specific WTP of Taiwan (Figure 4).

## DISCUSSION

This study presents an assessment of the potential clinical benefits and cost-effectiveness of PBT compared with SBRT in the treatment of inoperable advanced HCC. The literature on SBRT for small mHCC is extensive, but the information on the consequences of proton therapy for mHCC patients is very little. In particular, the information on cost-effectiveness data is limited.

Treatment of the base case population of mHCC patients resulted in an incremental cost per QALY gained of about NT$ 557, 907. The results also indicated that the incremental cost-effectiveness ratio could be considerably lower if patients at higher risk of severe toxicity were chosen for the treatment. The cost- effectiveness is thus highly dependent on the possibility of selecting appropriate risk patients for the therapy. Our results are consistent with other previous cost-effectiveness and the recent published systematic review of CEA studies, they reported that PBT offers promising cost-effectiveness for several cancers based on careful patient selection [21-22].

To determine whether a treatment is regarded as cost-effective or not is often depended on the threshold value for the cost effectiveness ratio. The WTP thresholds based on per capita gross domestic product (GDP), has been promoted by the World Health Organization’s Choosing Interventions in 2001 [23-24] and has been used as the threshold value per QALY gained in a number of recent cost-effectiveness studies. The WTP thresholds are higher than our base case result, which indicates that proton therapy used for inoperable advanced HCC patients with a risk of developing radiation-induced liver disease and with a lower radiation tolerance. Therefore, proton therapy is cost-effective for high-risk patients.

We tested the stability of the results with various sensitivity analyses, which showed that the results were robust. Several key parameters are impacted on the cost-effectiveness results. One is the direct medical cost of treating an individual patient. The new radiation technique may result in higher or lower healthcare expenditure for each patient treated. Although the direct medical costs of the SBRT and PBT paid as a treatment package which include the cost of physician office visits, radiation therapy treatment items (such as radiation fraction, computerized treatment planning, dosimetry and vertification film, new screening or diagnosis capacity that allows more targeted treatment etc), other health care costs for hospital days, treatment of severe toxicities and routine follow-up clinic visit are not included in the package. Therefore, the direct medical cost in progression state was higher in SBRT than PBT. Another parameter is the reduction of utility of patients in each healthcare state by 30% and 34.3 % severe toxicity rates caused by the PBT and SBRT. The reduced utility may effect on patients quality of life. Finally, the tumor size is also a parameter impacted on the clinical outcomes, which presented in terms of tumor control rate or overall survival. The median size of target tumor treated was larger in SBRT than that in PBT ( 7.2 cm vs 4.5 cm). Therefore, the overall survival reported in the PBT trial was longer than that in the SBRT trial (31 months vs 17 months). The longer survival will incur the increase of the direct medical costs used in patient’s life expectancy.

Since years of 2006, SBRT can be accepted as a safe modality for small tumor (<5 cm size) in many countries [25-27]. Inoperable large mHCC remains a therapeutic challenge, but modern radiation modalities are emerging for local therapy. Recently, SBRT and PBT was reported with a high local control rate for inoperable large HCC(> 5cm) from a single institution [10-12, 28-29]. However, these modern radiation therapies are associated with high economic costs. In particular, proton therapy is today only available in a limited center worldwide. The capital investment and operating facilities are large [30] and information about the potential clinical benefits and cost-effectiveness of the therapy is important for future decisions about reimbursement for new technology. Although the analyses presented in this study were based on only one center uncertain estimates, the result is still an evidence of the potential cost-effectiveness of proton therapy for incurable cancer which thus would support reimbursement and investments in this technology. The decision makers at our BNHI need to be aware of the economic burden of new technologies to assess the cost-effectiveness, balance the increasing healthcare budget, and meet expectations from patients and clinical practice.

This study has several limitations worth mentioning. First, our country currently has only one center for PBT. The lack of empirical comparative evidence to support the clinical data from two clinical trials imputed in our model may slightly influence the accuracy of the model. Second, the percentage of extra-hepatic spread and vascular invasion in patients was higher in the PBT trial than the SBRT trial. This may have some influence on the outcomes in the PBT group in terms of overall survival and progression-free survival. Third, the percentage of Child-Pugh class B liver function was higher among patients recruited in the PBT trial than the SBRT trial. This difference may also influence utility. However, the variation and wide ranges of parameters in the sensitivity analyses offset these limitations and the results are robust.
